# Characterization of hERG1 channel role in mouse colorectal carcinogenesis

**DOI:** 10.1002/cam4.72

**Published:** 2013-07-22

**Authors:** Antonella Fiore, Laura Carraresi, Angela Morabito, Simone Polvani, Angelo Fortunato, Elena Lastraioli, Angelo P Femia, Emanuele Lorenzo, Giovanna Caderni, Annarosa Arcangeli

**Affiliations:** 1 Department of Experimental and Clinical Medicine, Section of Internal Medicine and Oncology, University of FlorenceFlorence, Italy; 2 Istituto Toscano TumoriFlorence, Italy; 3 LIGeMA, CeSAL, University of FlorenceFlorence, Italy; 4 NEUROFARBA Department, Section of Pharmacology and ToxicologyFlorence, Italy; 5 Department of Biomedical Clinical and Experimental Sciences, University of FlorenceFlorence, Italy; 6 Laboratory of Cancer Genetics and Gene Transfer, Core Research Laboratory, Istituto Toscano Tumori (CRL-ITT), AOU CareggiFlorence, Italy

**Keywords:** Apc^min/+^ mice, azoxymethane, colorectal cancer, hERG1 channel, VEGF-A

## Abstract

The human *ether-à-go-go-related gene* (hERG)1 K^+^ channel is upregulated in human colorectal cancer cells and primary samples. In this study, we examined the role of hERG1 in colorectal carcinogenesis using two mouse models: adenomatous polyposis coli (Apc^min/+^) and azoxymethane (AOM)-treated mice. Colonic polyps of Apc^min/+^ mice overexpressed *mERG1* and their formation was reverted by the hERG1 blocker E4031. AOM was applied to either hERG1-transgenic (TG) mice, which overexpress hERG1 in the mucosa of the large intestine, or wild-type mice. A significant increase of both mucin-depleted foci and polyps in the colon of hERG1-TG mice was detected. Both the intestine of TG mice and colonic polyps of Apc^min/+^ showed an upregulation of phospho-Protein Kinase B (pAkt)/vascular endothelial growth factor (VEGF-A) and an increased angiogenesis, which were reverted by treatment with E4031. On the whole, this article assigns a relevant role to hERG1 in the process of *in vivo* colorectal carcinogenesis.

## Introduction

Colorectal cancer (CRC) is one of the most common cancers among men and women in developed and industrialized countries 1. Some of these tumors are hereditary 2,3, while the majority are sporadic and linked to environmental factors 4.

In the last few decades, CRC progression was deciphered as an ordered multistep process, which depends on a series of genetic changes, involving the lack of tumor-suppressor and DNA-repair genes accompanied by the activation of oncogenes 5. Each of these genetic aberrations leads to the accomplishment of specific morphological steps which lead the normal colonic mucosa to a true invasive carcinoma, through specific, ordered lesions 6.

Different mouse models, either genetically modified or chemically induced, have been generated to recapitulate the adenoma–carcinoma sequence that occurs in CRC, with the aim of better defining the molecular basis of the disease and identifying novel drugs for treatment.

The genetic mouse model mostly used for this purpose is represented by adenomatous polyposis coli (Apc^min/+^) mice, which carry a dominant heterozygous nonsense mutation at codon 850 of the mouse homologue of the human *APC* gene. Apc^min/+^ mice develop spontaneous multiple adenomas throughout the intestinal tract, mainly in the small intestine 7,8.

Chemically induced colorectal carcinogenesis in rodents represents one of the most used animal models of CRC. Azoxymethane (AOM), a 1,2-dimethylhydrazine metabolite 9,10, triggers colonic tumorigenesis in rodents 11, which almost totally reproduces the genetic and molecular alterations underlying CRC tumor progression 12, through a multistep process involving precancerous lesions such as mucin-depleted foci (MDF), adenomas, and cancers 10.

Among the genes whose expression is altered during the carcinogenetic process, those encoding ion channel and transporters are acquiring a relevant role in the last few years 13. In particular, K^+^ channels of the *ether-à-go-go* (EAG) family, mainly human *ether-à-go-go-related gene* (hERG1) 14 and EAG-1 15, were found to be overexpressed in several types of human cancers 13,16, including CRC 17–20. Moreover, the genes encoding either channels were detected in the crypts of murine colon, after carcinogen treatment 18, and an upregulation of oncogenic K^+^ ion channels (BK, Elk1, and EAG) was detected in the colon of Apc^min/+^ mice 21.

We tested the *in vivo* relevance of hERG1 channels during colorectal cancerogenesis by studying either Apc^min/+^ mice or AOM-treated mice. For this purpose, we produced hERG1-transgenic (TG) mice, which overexpress the *hERG1* gene in the intestinal mucosa.

## Materials and Methods

### Mouse strains and production of TG mice

Fabp-Cre mice were purchased from National Cancer Institute − Mouse Models of Human Cancers Consortium (NCI-MMHCC) 22 and Apc^min/+^ mice were obtained from The Jackson Laboratory (stock number: 002020).

The 10-kb vector-free XbaI DNA fragment was microinjected into the male pronucleus of fertilized eggs from FVB mice at LIGEMA, University of Florence, Italy, following standard procedures. TG mice were maintained in a heterozygous state in FVB background.

Animals were housed in plastic cages with a wire mesh providing isolation from the hygienic bed and were kept in temperature-, air-, and light-controlled conditions. They received food and water ad libitum. All experiments involving mice were performed in accordance with the criteria outlined in the Guide for the Care and Use of Laboratory Animals.

### AOM treatment

Two-month old mice, 12 TG, and six controls, maintained in a C57BL6/FVB mixed background, received intraperitoneal (IP) injections of AOM (10 mg/kg body weight) once a week for 6 weeks; in addition, three controls and six TG mice were treated with physiologic solution. Three months after the last injection, all animals were killed to evaluate tumorigenesis. The entire gastrointestinal tract was removed for dissection and flushed with phosphate buffered saline (PBS) to remove intestinal content. The intestine was opened longitudinally and washed extensively with PBS. Colon–rectum was fixed in 4% formaldehyde made in PBS for 24 h, after which the tissues were stained with methylene blue (0.1% for 10 min). The number of polyps was determined under a dissecting microscope (20× power field).

Aberrant crypt foci (ACF) were determined according to Bird 23. The same methylene blue-stained colons were then restained with high-iron diamine Alcian blue (HID-AB), to identify MDF as described in Ref. 24. MDF and ACF were identified under a microscope (400× magnification).

### E4031 treatment of Apc^min/+^ and TG mice

Apc^min/+^ mice received daily for 3 months IP injections of 20 mg/kg E4031 (TOCRIS, Bristol, U.K.) dissolved in sterile water; control Apc^min/+^ mice received buffered saline only. After 3 months, animals were killed by cervical dislocation. The entire gastrointestinal tract was removed for dissection and flushed with PBS to remove intestinal content. The colon–rectum was opened longitudinally and washed extensively with PBS, fixed in 4% buffered formaldehyde for 24 h and then stained with methylene blue. The number of polyps was determined under a dissecting microscope (20× power field).

TG mice received daily for 14 days IP injections of 20 mg/kg E4031 (TOCRIS) dissolved in sterile water; control mice received buffered saline only. After 14 days, animals were killed by cervical dislocation. The entire gastrointestinal tract was removed for dissection and flushed with PBS to remove intestinal content. The organ was opened longitudinally and washed extensively with PBS, fixed in 4% buffered formaldehyde for 24 h and embedded in paraffin. Tissue sections (7 μm) were cut from blocks using a microtome (Leica RM2125/RM2125RT, Nussloch, Germany). Immunohistochemistry (IHC) using antivascular endothelial growth factor (VEGF-A) antibody was performed to evaluate differences between control and treated TG mice.

### RNA extraction, reverse transcription, and real-time quantitative PCR

RNA was extracted from different tissues of wild-type (WT), TG, and Apc^min/+^ mice using TRIzol reagent (Invitrogen, Groningen, the Netherlands) according to the manufacturer's protocol. cDNA was obtained from 1 to 2 μg of RNA using 200 U reverse transcriptase SuperScript II (Invitrogen), plus 500 μmol/L each of deoxyribo-nucleotide triphosphate (dNTP) and 15 ng/μL of random primers, in a 20 μL final reaction volume, for 50 min at 42°C and 15 min at 70°C. cDNA synthesis was monitored by PCR with β-*actin* primers.

*hERG1*, *mERG1*, and *myosin, heavy polypeptide 11, smooth muscle* (*myh11*) mRNA quantification by real-time quantitative PCR (RT-qPCR) were performed on 2 μL of cDNA (diluted 1:4) using the 7500 Fast RT-PCR System and the SYBR Green Master Mix Kit (Applied Biosystems; Foster City, CA). The *β*-*actin* gene was used as RT-qPCR reference gene. The primer sequences for *hERG1* were: 5′-CTCACCGCCCTGTACTTCAT-3′ forward primer and 5′-GCTCCCCAAAGATGTCATTC-3′ reverse primer; for *β*-*actin*: 5′-GGGGTGTTGAAGGTCTCAAA-3′ forward primer and 5′-GATCTGGCACCACACCTTCT-3′ reverse primer; for *mERG1*: 5′-GGACCTGCTTACTGCCCTCT-3′ forward primer and 5′-GGACGGGCATATAGGTTCAG-3′ reverse primer; and for *myh11*: 5′-CGACAGGCTAGGGATGAGAG-3′ forward primer and 5′-GCTCTCCAAAAGCAGGTCAC-3′ reverse primer. The relative gene expression was calculated applying the Pfaffl analysis method 25.

### Immunohistochemistry

IHC was performed on 7-μm sections of tissues fixed in 4% formalin and embedded in paraffin, mounted on positively charged slides. After dewaxing and blocking endogenous peroxidases, sections were treated with proteinase K (Roche, Mannheim, Germany; 5 μg/mL in PBS) and UltraVBlock solution (LabVision, Fremont, CA) containing 0.1% Triton X-100, and then incubated with the following primary antibodies: anti-hERG1 monoclonal antibody 20 (dilution 1:200 in PBS-UltraVBlock), anti-VEGF-A (Santa Cruz Biotechnology, Santa Cruz, CA) (dilution 1:100 in PBS-UltraVBlock), anti-CD34 (Cedarlane, Burlington, NC) (dilution 1:100 in PBS-UltraVBlock) overnight at 4°C, and anti-phospho-Protein Kinase B (pAkt) (Santa Cruz Biotechnology) (dilution 1:100 in PBS-UltraVBlock) for 1 h at 37°C. For pAkt detection, antigen retrieval was carried out by heating slides in a microwave for 10 min at 600 W in citrate buffer (pH 6). For CD34 detection, antigen retrieval was carried out by irradiating the slides in a microwave for 20 min at 700 W in citrate buffer (pH 7.8).

Immunostaining was carried out using a commercially available kit (PicTure Plus kit; Zymed, San Francisco, CA). After extensively washing with PBS, color was developed by incubating the slides with the 3,3′-diamino-benzidine chromogen solution for 2–5 min or until acceptable color intensity had been reached. Slides were then counterstained with Mayer's hematoxylin and mounted using Entellus mounting medium. Images were acquired on a Leica DM 4000B microscope with a Leica DFC 320 camera using Leica Win software (Leica Microsystems; Milan, Italy).

### Statistical analysis

Data obtained from AOM-treated mice were reported as mean ± standard error of the mean (SEM) and analyzed by Mann–Whitney *U* test. A *P*-value <0.01 (*) was considered statistically significant.

Data obtained from vessel and total vascular area count of WT, TG, and Apc^min/+^ mice were reported as mean ± SEM and analyzed by Mann–Whitney *U* test. *P*-values <0.05 and <0.01 were considered statistically significant.

Data obtained from VEGF-A and pAkt expressions were analyzed by Mann–Whitney *U* test. *P*-values <0.05 and <0.01 were considered statistically significant.

Data obtained from RT-qPCR experiments to detect *mERG1* expression in TG and WT mice were reported as mean ± SEM and analyzed by Mann–Whitney *U* test (*n*: four WT mice and five TG mice). A *P*-value <0.05 (*) was considered statistically significant.

## Results

### Role of hERG1 in colonic polyp development of Apc^min/+^ mice

We studied the role of hERG1 channels in CRC tumorigenesis, using Apc^min/+^ mice as a model. On the basis of previous observations 26 indicating that the transcript encoding the murine homologue of the *hERG1* gene, *mERG1*, was expressed in the colon of Apc^min/+^ mice, we first determined *mERG1* expression levels in various tracts of the intestine of such mice, by RT-qPCR.

**Figure 1:**
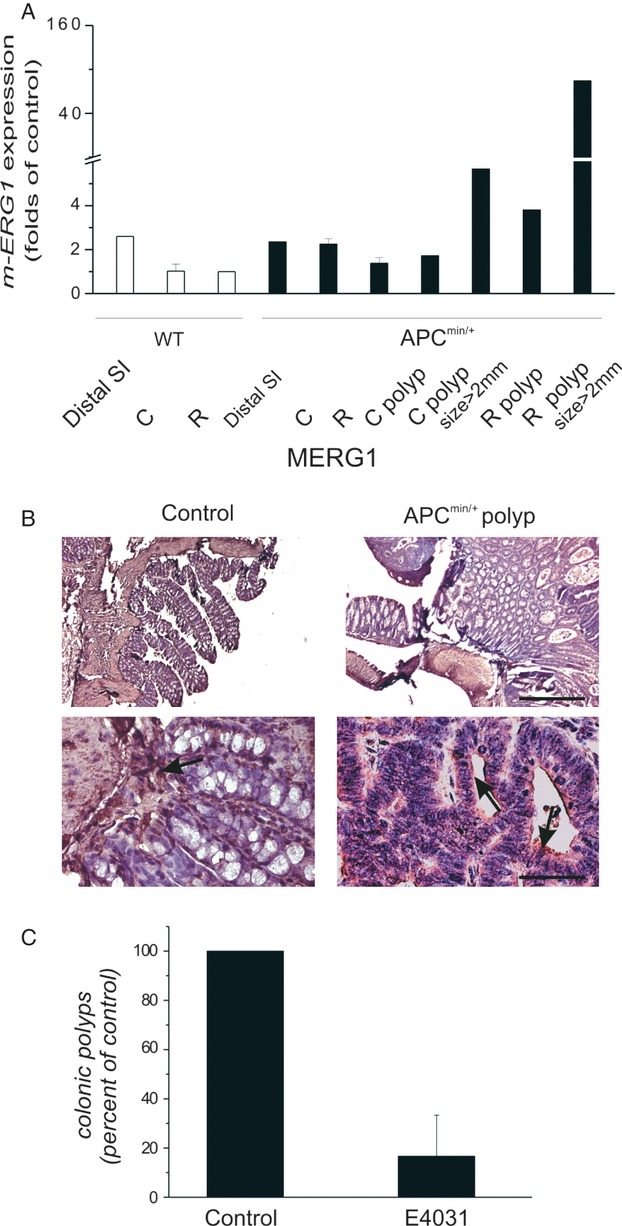
Expression and role of *mERG1* in Apc^min/+^ mice. (A) Analysis of *mERG1* expression by RT-PCR in small and large intestine of WT and Apc^min/+^ mice and in colonic and rectal polyps developed in Apc^min/+^ mice after a normalization for mouse *myh11*, characteristic of myofibroblasts and smooth muscle cells, to detect the only *mERG1* epithelial expression^27^. Distal SI, distal small intestine; C, colon; R, rectum; C polyp, colonic polyp; R polyp, rectal polyp. Data relative to colon and rectum derived from two different experiments, each carried out in triplicate, are reported as the mean ± SEM and were calibrated to the expression levels determined in the rectum of WT mice. Data relative to distal small intestine and polyps derived from a single experiment, carried out in triplicate, are reported as the mean, and were calibrated to the expression levels determined in the rectum of WT mice. (B) mERG1 expression in control (WT) and Apc^min/+^ polyps was evaluated by IHC. An anti-hERG1 monoclonal antibody was used as detailed in Material and Methods. Upper panels: 50× magnification, bar: 200 μm; lower panels: 400× magnification, bar: 20 μm. (C) The number of colonic polyps obtained after E4031 treatment of Apc^min/+^ mice. Four 1-month-old Apc^min/+^ mice received daily IP injections of E-4031 for 3 months, while two Apc^min/+^ mice received buffered saline only. After death, the number of polyps that developed in colon of Apc^min/+^ mice was determined under a dissecting microscope (20× power field). Data were expressed as mean ± SEM.

We found that the colon and rectum of Apc^min/+^ mice showed an increase in *mERG1* expression, compared to that in WT mice. Such increase was more evident in colonic and rectal polyps, which spontaneously develop in these mice. Interestingly, *mERG1* expression increased along with the size of polyps (Fig. 1A). The mERG1 protein was also detected by IHC in colonic polyps of Apc^min/+^ mice (Fig. 1B). Polyps displayed a high mERG1 expression in adenomatous epithelial cells (see arrows in Fig. 1B, right panel), while colonic samples of WT mice showed only a faint signal in the stroma (Fig. 1B, left panel).

When Apc^min/+^ mice were treated with the specific hERG1 blocker E4031, daily for 3 months, such long-term mERG1 current inhibition 28–32, that, in agreement with a previous paper 33, did not lead to drug-induced torsades de pointes in E4031-treated mice, produced an impairment in colonic lesion development (Fig. 1C). No effects on the number of polyps in the small intestine were observed. Consistently, no overexpression of the *mERG1* transcript was detected in the small intestine of Apc^min/+^ compared to WT mice (Fig. 1A, left most bar).

### Effects of AOM treatment in hERG1 TG mice

We then investigated the possible role of hERG1 in a chemically induced mouse model of CRC, utilizing AOM as carcinogen, and treating either WT or genetically modified mice, hERG1-TG mice, which overexpress the *hERG1* gene in the intestinal mucosa.

**Figure 2:**
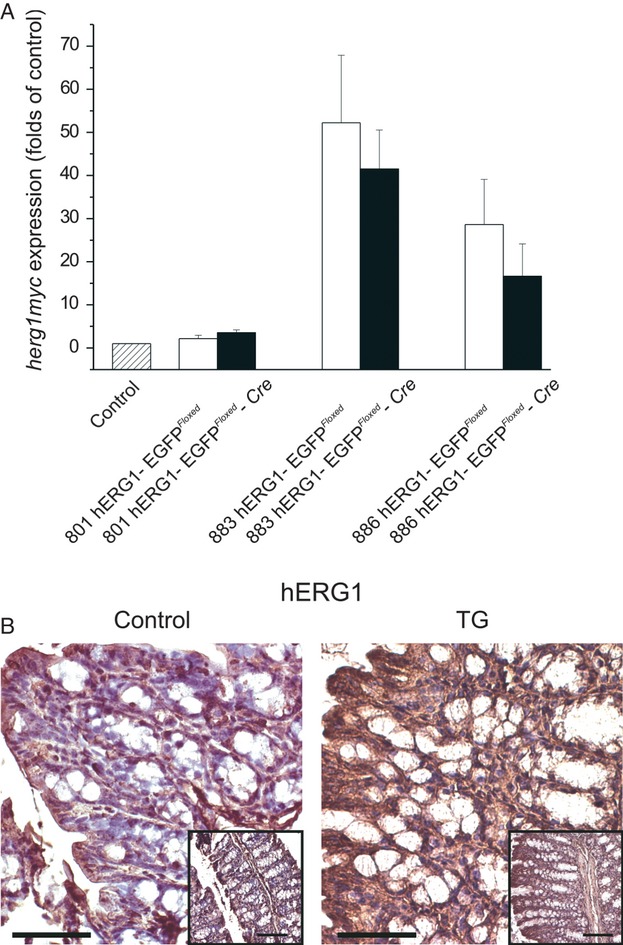
Characterization of hERG1 expression in TG mice. (A) Analysis of *hERG1myc* expression by RT-PCR in colon–rectum of WT mice (left bar), hERG1-EGFP^Floxed^ mice (white bars), and hERG1-EGFP^Floxed^-Cre mice (black bars), respectively from 801, 883, and 886 transgenic line. Data, each carried out in triplicate, are reported as the mean ± SEM and were calibrated to the expression levels determined in the colon–rectum of WT mice. (B) An immunohistochemical analysis was carried out in colon–rectum of control and TG mice, using an anti-hERG1 monoclonal antibody, as detailed in Material and Methods, to evaluate hERG1 expression and confirm the presence of the transgene. Magnification 400×, bar: 20 μm; inset: 200× magnification, bar: 50 μm.

The TG mouse model was developed by us, and the procedure we adopted is detailed in the supplementary information section. Briefly, the *hERG1* cDNA, tagged with the myc epitope and a poly-histidine (His) flag at the protein C-terminal, was put under the control of the human β-*actin* minimal promoter, with an intercalated floxed reporter *EGFP* gene, which should block transgene transcription. However, hERG1-EGFP^Floxed^ mice expressed the *hERG1* transcript both in the colon (Fig. 2A, white bars) and in the liver (Fig. S1D), even in the absence of Cre-mediated recombination (compare white and black bars in Fig. 2A). Hence, a transcriptional readthrough phenomenon occurred, and no further significant increase in *hERG1* expression was triggered by Cre, for example after mating hERG1-EGFP^Floxed^ mice with Fabp-Cre mice. The latter mice express the Cre recombinase under the control of the Fabp promoter, hence in the whole digestive tract 22. Therefore, hERG1-EGFP^Floxed^ mice, due to the transcriptional control exerted by the β-*actin* promoter, can be considered to overexpress the *hERG1* transcript ubiquitously. Both the *hERG1* transcript (Fig. 2A) and the hERG1 protein (Fig. 2B) were overexpressed in TG mice (either hERG1-EGFP^Floxed^ or hERG1-EGFP^Floxed^ -Cre) belonging to different TG lines (801, 883, and 886), compared to WT mice. In TG mice, hERG1 expression was strongly detectable in colonic epithelial cells and not limited to the stroma and myofibroblasts, as in WT mice. A slight, significant difference in *mERG1* expression in TG compared to WT mice (1.88 ± 0.2 *mERG1* expression in TG vs. 1.16 ± 0.1 *mERG1* expression in WT; *P *=* *0.03, Mann–Whitney *U* test) was detected, even if such *mERG1* expression increment in TG mice resulted to be very minimal when compared to the artificial *hERG1* expression. The generated TG mice did not show any apparent phenotype, even at old ages, and presented a normal life span.

**Figure 3:**
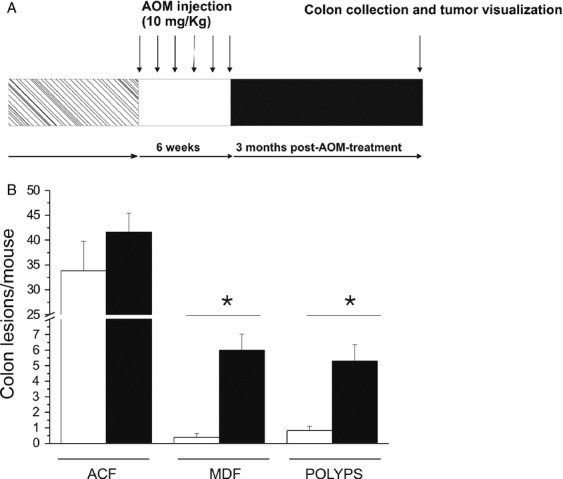
Effect of AOM treatment in hERG1 TG mice. (A) Outline of AOM treatment: six control mice and 12 TG mice, maintained in a C57Bl6/FVB mixed background, received, at 2 months after birth, IP injections of AOM (10 mg/kg body weight) once a week for 6 weeks and were killed 3 months after the last injection. (B) The number of ACF, MDF, and polyps that developed in control (white bars) and TG (black bars)-treated mice was determined. Data were expressed as mean ± SEM. Statistical analysis was conducted using the Mann–Whitney *U* test (*significantly different with a *P*-value of <0.01).

Both TG and WT mice were treated with AOM (or physiologic saline), according to the schedule in Figure 3A, and the occurrence of colonic lesions was analyzed 3 months after the last injection. The macroscopic inspection of the large intestine of treated mice revealed, in the colon of AOM-treated TG mice, the presence of polyps that was, on the contrary, only barely detectable in WT mice (Fig. 3B). No lesions were observed in the large intestine of mice injected with physiologic saline. After staining the large intestine with methylene blue, the number of carcinogen-induced ACF, and after restaining with HID-AB, that of MDF was determined. A statistically significant increase in the number of MDF lesions in TG mice compared to WT mice was detected (Fig. 3B). The increase in MDF lesions paralleled the increased number of polyps, as evidenced by macroscopic inspection. No significant difference was detected in the number of ACF between TG and WT mice.

### TG and Apc^min/+^ mice overexpress pAkt and VEGF-A in the epithelial lining of the large intestine

Finally, we tried to decipher whether a common molecular mechanism could underline the effect of hERG1 overexpression in the process of colorectal carcinogenesis, as evidenced in either the genetic (Apc^min/+^) or chemical (AOM treated) mouse model. In different types of cancers, including CRC 20,34,35, hERG1 is functionally linked to the pathway that promotes the secretion of the VEGF-A, hence contributing to tumor angiogenesis 36.

**Figure 4:**
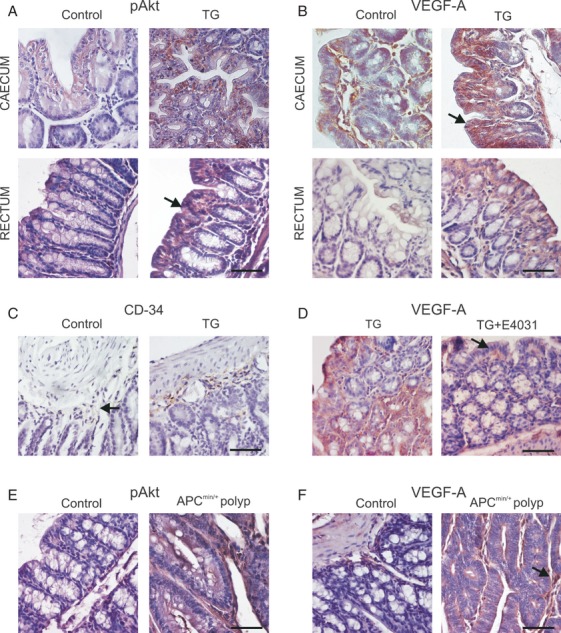
pAkt and VEGF-A expressions in hERG1 TG and Apc^min/+^ mice. (A) IHC experiments were performed for pAkt in cecum and rectum of control and TG mice. Experiments were carried out using an anti-pAkt antibody (Santa Cruz, dilution 1:100), following the same protocol reported in Materials and Methods. 400× magnification, bar: 20 μm. (B) Representative pictures of VEGF-A expression for both control and TG mice are reported. An anti-VEGF-A polyclonal antibody (Santa Cruz, dilution 1:100) was used as detailed in Materials and Methods. In both groups, we evaluated VEGF-A expression in the cecum and rectum of animals of different ages (3 and 6 months). Experiments performed on 6-month-old mice were reported, but the same results were obtained with 3-month-old mice. Magnification 400×, bar: 20 μm. (C) IHC using anti-CD34 monoclonal antibody (Cedarlane Laboratories, dilution 1:100) was performed, as detailed in Materials and Methods. Magnification 400×, bar: 20 μm. (D) Representative pictures of IHC experiments performed with anti-VEGF-A antibody are shown for both control TG mice and E4031-treated TG mice (addressed as E4031). Magnification 400×, bar: 20 μm. (E and F) Immunohistochemical staining for pAkt (left panels) and VEGF-A (right panels) in control and Apc^min/+^ polyps. Magnification 400×, bar: 20 μm.

On the basis of this assumption, we analyzed the expression of both pAkt (i.e., the kinase which regulates *VEGF* expression 37) and VEGF-A in the large intestine of TG and in the mERG1-expressing polyps of Apc^min/+^ mice. A higher expression of both pAkt (Fig. 4A) and VEGF-A (Fig. 4B) was detected in the proximal colon and rectum of TG compared to WT mice. Similarly and consistent with previous reports 38,39, a clear pAkt (Fig. 4E) and VEGF-A (Fig. 4F) immunostaining was detected in the polyps of Apc^min/+^ mice with significantly higher levels compared with control mice (Table 1). In both TG mice and Apc^min/+^ polyps, VEGF-A displayed a peculiar expression pattern, different from that observed in control mice. In fact, VEGF-A expression was not limited to the stroma, but was significantly present in the cells of the epithelial lining. The increased expression of VEGF-A in TG mice and Apc^min/+^ polyps was accompanied by a significant increase in angiogenesis, evaluated as microvessel density and total vascular area measured after staining with an anti-CD34 antibody (Fig. 4C and Table 2). Control mice showed smaller vessels mainly localized in the *muscolaris propria*, while TG mice and Apc^min/+^ polyps were characterized by larger vessels with a less ordinate distribution.

**Table:** VEGF-A and pAkt expressions in WT, TG, and Apc^min/+^ mice evaluated by the percentage of positively immunostained cells

	WT mucosa (FVB)			TG mucosa					WT mucosa (C57BL/6)	APC^min/+^ mucosa	APC^min/+^ polyps
3 months	6 months	3 months	6 months	6 months	6 months	6 months
VEGF-A	45%	30%	70%	70%	1%	5%	60%
			NS	*P* < 0.01*			*P* < 0.01** *P* < 0.01***
pAkt	25%	10%	60%	60%	10%	10%	30%
			*P* < 0.05*	*P* < 0.05*			*P* < 0.05** *P* < 0.05***

WT, wild-type mice; TG, hERG1-transgenic mice; NS, nonsignificant. Statistical analysis: Mann–Whitney *U* test; *n* = 6 microscopic fields at 200× magnification.

*P* = TG mice versus corresponding age-matched WT (FVB) mice.

*P* = 6-month-old Apc^min/+^ polyps versus 6-month-old WT mucosa (C57BL/6).

*P* = 6-month-old Apc^min/+^ polyps versus 6-month-old Apc^min/+^ mucosa.

**Table:** Vessel and total vascular area count in WT, TG, and Apc^min/+^ mice

	WT mucosa (FVB)			TG mucosa					WT mucosa (C57BL/6)	APC^min/+^ mucosa	APC^min/+^ polyps
3 months	6 months	3 months	6 months	6 months	6 months	6 months
Number of vessels	5.6 ± 0.7	3.6 ± 0.8	10.1 ± 1.1	8.6 ± 1.6	3.9 ± 0.3	4.7 ± 0.6	15.9 ± 3.3
			*P* < 0.01*	*P *<* *0.01*			*P *<* *0.05** *P *<* *0.05***
Total vascular area (mm^2^/microscopic field)	3.4 ± 0.6	2 ± 0.4	3.4 ± 0.6	5 ± 1.2	2.8 ± 0.6	3 ± 0.5	21.6 ± 7.5
			NS	*P *<* *0.05*			*P *<* *0.01** *P *<* *0.01***

WT, wild-type mice; TG, hERG1-transgenic mice; NS, nonsignificant. Data are reported as mean ± SEM; statistical analysis: Mann–Whitney *U* test; *n *=* *12 microscopic fields at 200× magnification.

*P* = TG mice versus corresponding age-matched WT (FVB) mice.

*P* = 6-month-old Apc^min/+^ polyps versus 6-month-old WT mucosa (C57BL/6).

*P* = 6-month-old Apc^min/+^ polyps versus 6 months-old Apc^min/+^ mucosa.

Finally, we verified whether the upregulation of VEGF-A in TG mice was directly linked to a higher hERG1 activity and, hence, could be reverted by treatment with the hERG1 blocker E4031. Indeed, treatment of TG mice with E4031 for 2 weeks, led to a significant decrease in VEGF-A staining (Fig. 4D). This indicates that hERG1 channels are not only overexpressed in TG mice, and drive VEGF-A secretion and an increased angiogenesis but they are active and their activity is more or less directly responsible for the VEGF-A-enhanced production observed in these mice.

## Discussion

Substantial evidence indicates that cancer can be partially attributed to ion channel malfunction. Numerous studies included hERG1 in the list of ion channels mis/overexpressed in cancer cells, where it plays the role of regulator of tumor cell proliferation and progression 40,41. In this article, we analyzed the role of hERG1 in colorectal carcinogenesis *in vivo*, using either genetic (Apc^min/+^ mice) or chemical (AOM treated) models of CRC. In both models, we found a relevant role of hERG1 channels, which could be traced back to a hERG1-dependent control of angiogenesis.

Colonic and rectal polyps, which spontaneously develop in Apc^min/+^ mice, showed an evident overexpression of the murine homologue of *hERG1*, *mERG1* and the long-term treatment of Apc^min/+^ mice with the specific hERG1 blocker, E-4031, suppressed polyp formation in the large intestine. It is known that the main function of *Apc* is to degrade cytosolic levels of β-catenin, whose dysregulation is considered a major cause of tumor development. As previous studies from Carlos Munoz's laboratory showed that β-catenin increased the hERG1 protein levels within the oocyte cell membrane 42, the increased hERG1 channel activity detected in the polyps of Apc^min/+^ mice could be attributed to the overexpression of β-catenin, widely described in this animal model 43. It is worth noting that, at difference from what happens in the small intestine, the loss of function of *Apc* is not sufficient per se to trigger the development of tumors in the colon, where adjunctive genetic events are required for the transition from microadenomas to macroscopic tumors to be accomplished 44. Our data could suggest considering hERG1 as one factor which cooperates with Apc loss to trigger colorectal tumor progression.

This conclusion is further supported by data obtained in carcinogen-treated mice. In this case, in order to analyze the role of hERG1, we generated a TG mouse model overexpressing hERG1. On the bases of the construct we used, we expected to obtain a conditional hERG1-expressing mouse. On the contrary, due to a readthrough phenomenon, the mice we generated showed a ubiquitous expression of hERG1. Even when hERG1 was expressed at high levels, the hERG1-TG mice did not show any overt phenotype and presented a normal life span. Hence, hERG1 overexpression per se is not life-threatening and does not induce tumor development. Although hERG1-TG mice did not develop spontaneous tumor, they displayed an accelerated process of tumorigenesis, when treated with AOM, as witnessed by an increased number of preneoplastic lesions (mainly MDF) and polyps in the colon. It is worth noting that MDF, that is dysplastic lesions, characterized by a defective mucin production, are considered precursors of CRC both in humans 8,45,46 and in experimental models 24. Our finding that the concomitant hERG1 and, probably transgene induced, mERG1 overexpression in the large intestine increases the number of AOM-induced MDF and polyps, strongly indicates that an upregulation of hERG1 accelerates the process of colorectal carcinogenesis, further stressing the role of hERG1 as a progression gene in CRC.

On the whole, the *hERG1* gene can be considered a “tumor progression” gene, as it strongly cooperates with genetic (loss of the tumor-suppressor gene *Apc*) or environmental (chemical carcinogen) factors in triggering CRC progression.

Finally, we provided evidence that the role of hERG1 in CRC carcinogenesis can be traced back to its role in the signalling pathways, which regulate VEGF-A secretion and neoangiogenesis. In CRC cells, hERG1 channels regulate pAkt, through the formation, on the plasma membrane, of a macromolecular complex between hERG1, the β1 integrin, and the p85 subunit of phosphatidyl inositol-3-kinase, which leads to the activation of Akt (O. Crociani et al., pers. comm.). Consistently, we found an upregulation of pAkt and VEGF-A expression in both Apc^min/+^ polyps and hERG1-TG mice. In the latter, the increased VEGF-A expression causes an increased angiogenesis, which was reverted by blocking hERG1 with a specific blocker. Hence, VEGF-A expression *in vivo* depends on hERG1 activity. Therefore, when hERG1 is aberrantly overexpressed, VEGF-A secretion and angiogenesis are concomitantly upregulated; through the increased angiogenesis, hERG1 activity may influence tumor development and progression.

Taken together, data reported in this article indicate a significant role of hERG1 in colorectal carcinogenesis *in vivo*, confirming indications, obtained in the human setting, that an early overexpression of the hERG1 gene marks those precancerous lesions of the upper gastrointestinal tract which undergo malignant progression 47. Moreover, data here provided further stress the inclusion of hERG1 blockers in the treatment of CRC.

## Supporting Information

Figure S1 (A) Schematic representation of *hERG1myc* conditional expression vector, including location of specific primers (1–7): 1–2 utilized for PCR genotyping specific for the *hERG1myc* transgene; 3–4 used to detect transcriptional readthrough phenomenon, by amplification of a cDNA fragment spanning from the 3′ end of the *egfp* (primer 3) to the 5′ end of *hERG1myc* (primer 4); 5– 4 used to detect recombination in double transgenic mice; 6–7: utilized for *hERG1myc* mRNA quantification by RTqPCR. Restriction sites and the probe used in the Southern blot analysis are indicated. The β*-actin egfp hERG1* construct was assembled in pBluescript SK plasmid. The complete vector contains the 4.3 kb human β-*actin* promoter plus intron [48] and the SV40 polyadenylation sequence, both derived from the β-actpA plasmid, kindly provided by Dr. S. Aparicio (BC Cancer Agency, Vancouver, British Columbia, Canada), a floxed stop cassette and the *hERG1-myc 6xhis* cDNA. The stop cassette is represented by the reporter gene *egfp* followed by an SV40 polyadenylation sequence, obtained by amplification from the pEGFP C1 plasmid (Clontech, Palo Alto, CA) and floxed by two loxP present in the D11 loxP plasmid (gift from Dr. S. Aparicio). It was cloned in the unique HindIII site of the β-actpA plasmid between the β-*actin* promoter and the *hERG1-myc 6xhis* cDNA. (B) Southern blot analysis was carried out on 10 lg of genomic tail DNA extracted from mice of each transgenic line. Genomic DNA, digested with HindIII, was transferred to Hybond N+ membrane (GE Healthcare, Buckinghamshire, U.K.) and tested with a 32P-labeled 1.5 kb probe corresponding to a EGFP fragment, as indicated in (A). Transgene copy number was estimated comparing the intensity of DNA band of transgenic animals to a standard of 1, 10, and 50 copies of injected DNA fragment using the ImageJ software. (C) Mice were genotyped by PCR analysis of genomic tail DNA. Briefly, 0.5 cm of mice tails were digested O/N at 55°C with 100 mg/mL proteinase K in lysis buffer (50 mmol/L TrisHCl pH 8, 50 mmol/L EDTA pH8, 1% SDS, 10 mmol/L NaCl); after phenol/ chloroform purification and ethanol precipitation, genomic DNA was resuspended in sterile water. Alternatively, genomic DNA was extracted using a Chelex protocol by incubation of a 0.2 cm of mice tails in 150 lL of 10% (w/v in water) Chelex 100 resin (BIO-RAD, Milan, Italy) for 4 h at 56°C and 30 min at 98°C. The presence of the transgene was checked on mouse tail genomic DNA by amplification of a 350 bp fragment (upper panel) spanning the myc-6xhis epitope region with the forward 5′- TTTAAACTTAAGCTGGAGAC-3′ and reverse 5′- CTACAGTGCTGTGACCAC-3′ primers (as indicated in 1A) in the following PCR conditions: denaturation at 94°C for 2 min, 35 cycles at 94°C for 40 sec, 54°C for 1 min, 72°C for 1 min, and a final extension cycle at 72°C for 5 min. Amplification of the control gene, interleukin- 2, by PCR (lower panel) was systematically performed on DNA to check the integrity of genomic DNA extracted, using the following primer pair: 5′-CTAGGCCACAGAATTGAAAGATCT- 3′ and 5′-GTAGGTGGAAATTCTAGCATCATCC- 3′ and applying the following PCR conditions: denaturation at 94°C for 2 min, 35 cycles at 94°C for 1 min, 60°C for 1 min, 72°C for 1 min and a final extension cycle at 72°C for 3 min. (D) hERG1-EGFPFloxed mice and WT mice were killed and livers were analyzed by RT-PCR to evaluate *hERG1- myc* mRNA. The analysis of *hERG1myc* expression levels in liver of WT mice (white bar) and hERG1-EGFPFloxed mice (black bars) respectively from 801, 883, and 886 transgenic line is shown. Data are reported as mean _ SEM with normalization to WT liver. (E) A readthrough phenomenon was identified by the sequencing of a cDNA fragment, amplified with primer 3: 5′- ACAACCACTACCTGAGCAC-3′ and primer 4: 5′-ATGA TGGTGTCCAGGAAG-3′ (as indicated in [A]), spanning from the 3′ end of the *egfp* to the 5′ end of *hERG1myc* of the construct. Bold: *egfp* sequence; cursive: polyA SV40 sequence; underscored: loxP sequence; bold and underscored: *hERG1myc* sequence.

Figure S2 (A) hERG1-EGFPFloxed mice were mated with Fabp4xat-132Cre (Cre) mice. In the latter mice, the Cre recombinase should be expressed, although not exclusively, in the intestinal epithelium. Reverse transcription PCR showed mRNA expression of *cre* in different large intestine segment of hERG1-EGFPFloxed-Cre double transgenic (DT) mice. Amplification of *cre* was performed with *Platinum* _ *PCR SuperMix* on 2 lL of cDNA derived from cecum, colon, and rectum of DT mice and with a specific primer pair: 5′-ACCAGCCAGCTATCAACTCG-3′ and 5′-TTACATTGGTCCAGCCACC-3′ and applying the following PCR conditions: denaturation at 94°C for 2 min, 35 cycles at 94°C for 1 min, 60°C for 1 min, 72°C for 1 min, and a final extension cycle at 72°C for 3 min. (B) The lack of a significant difference in *hERG1* expression in hERG1-EGFPFloxed-Cre compared to hERG1- EGFPFloxed mice was also confirmed at the protein level: an immunohistochemical analysis was carried out in colon-rectum of hERG1-EGFPFloxed and DT mice, employing anti-Myc antibody (anti-myc 9E10; Santa Cruz Biotechnology), dilution 1:100 in PBS-UltraVBlock). No gross difference in the amount of Myc staining could be detected in DT compared to hERG1-EGFPFloxed mice: hERG1-EGFPFloxed as well as DT mice showed myc expression in the stroma and in the epithelial lining. (C) hERG1-EGFPFloxed-Cre double transgenic genomic DNA was tested by end-point pcr to verify the presence of Cre mediated recombination in mice. DNA extracted from colon and rectum of WT and hERG1-EGFPFloxed- Cre double transgenic mice was amplified with the 5: 5′- AGGATCAGTCGAAATTCAAG-3′ and 4: 5′-ATGATGGTGTCCAGGAAG- 3′ primers and applying the following PCR conditions: denaturation at 94°C for 2 min, 35 cycles at 94°C for 40 sec, 52°C for 1 min, 72°C for 1 min, and a final extension cycle at 72°C for 5 min. Such end point PCR failed to detect recombination in hERG1-EGFPFloxed-Cre double transgenic mice (data not shown); on the other hand, PCR analysis performed on genomic DNA extracted from tails of hERG1-EGFPFloxed- CMVCre mice revealed the correct transgene recombination in the presence of Cre.
