# Supplementary material for: Characterization of hERG1 channel role in mouse colorectal carcinogenesis
Source: Cancer Med. 2013 Jul 22;2(5):583–94. doi: 10.1002/cam4.72 (PMC3892791; doi:10.1002/cam4.72)
Supplement: Figure S1 — (A) Schematic representation of hERG1myc conditional expression vector, including location of specific primers (1–7): 1–2 utilized for PCR genotyping specific for the hERG1myc transgene; 3–4 used to detect transcriptional readthrough phenomenon, by amplification of a cDNA fragment spanning from the 3′ end of the egfp (primer 3) to the 5′ end of hERG1myc (primer 4); 5– 4 used to detect recombination in double transgenic mice; 6–7: utilized for hERG1myc mRNA quantification by RTqPCR. Restriction sites and the probe used in the Southern blot analysis are indicated. The β-actin egfp hERG1 construct was assembled in pBluescript SK plasmid. The complete vector contains the 4.3 kb human β-actin promoter plus intron [48] and the SV40 polyadenylation sequence, both derived from the β-actpA plasmid, kindly provided by Dr. S. Aparicio (BC Cancer Agency, Vancouver, British Columbia, Canada), a floxed stop cassette and the hERG1-myc 6xhis cDNA. The stop cassette is represented by the reporter gene egfp followed by an SV40 polyadenylation sequence, obtained by amplification from the pEGFP C1 plasmid (Clontech, Palo Alto, CA) and floxed by two loxP present in the D11 loxP plasmid (gift from Dr. S. Aparicio). It was cloned in the unique HindIII site of the β-actpA plasmid between the β-actin promoter and the hERG1-myc 6xhis cDNA. (B) Southern blot analysis was carried out on 10 lg of genomic tail DNA extracted from mice of each transgenic line. Genomic DNA, digested with HindIII, was transferred to Hybond N+ membrane (GE Healthcare, Buckinghamshire, U.K.) and tested with a 32P-labeled 1.5 kb probe corresponding to a EGFP fragment, as indicated in (A). Transgene copy number was estimated comparing the intensity of DNA band of transgenic animals to a standard of 1, 10, and 50 copies of injected DNA fragment using the ImageJ software. (C) Mice were genotyped by PCR analysis of genomic tail DNA. Briefly, 0.5 cm of mice tails were digested O/N at 55°C with 100 mg/mL proteinase K in lysi [file cam40002-0583-sd1.pdf]

A

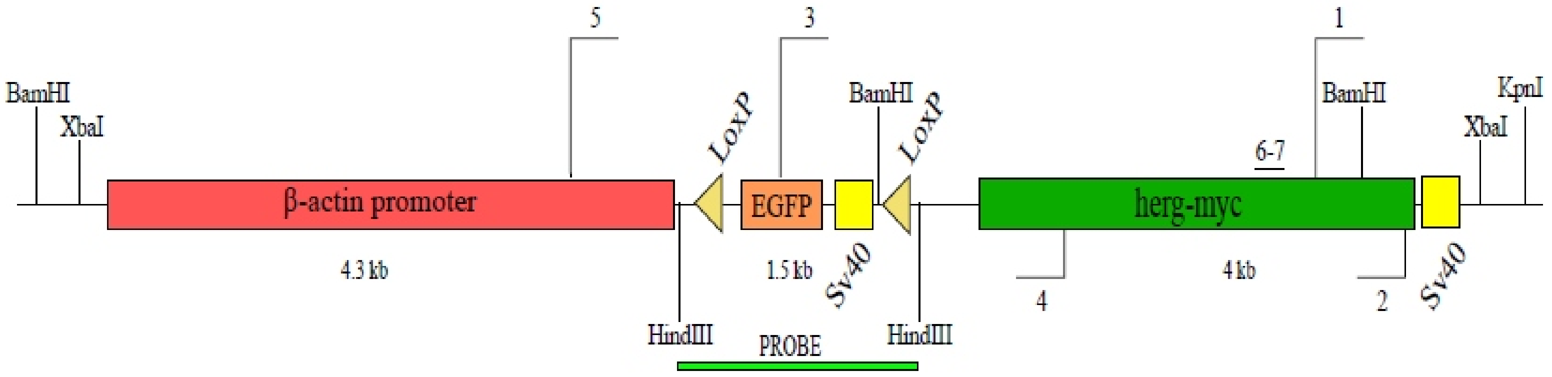


B

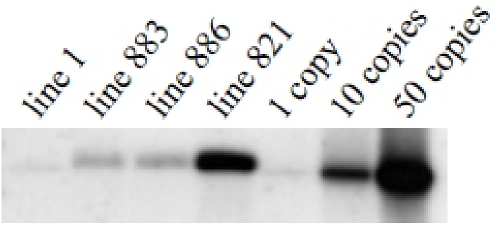


C

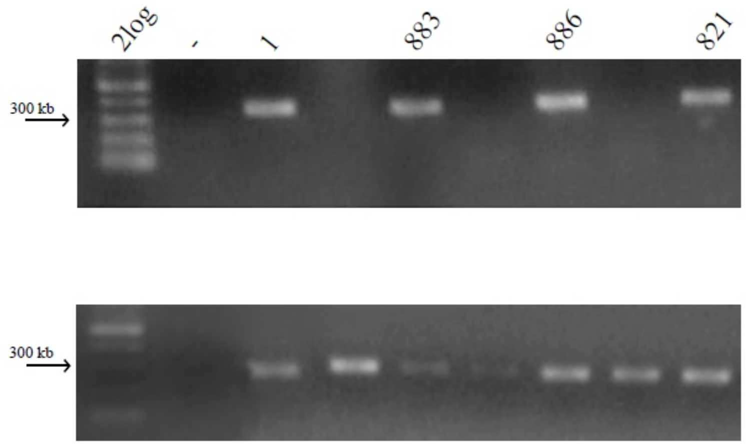


D

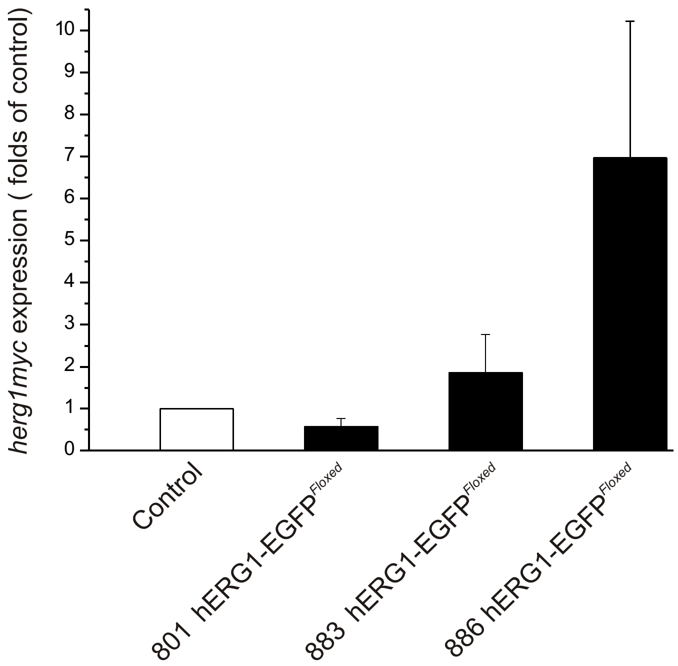


E

**CCCAACGAGAAGCGCGATCACATGGTCCTGCTGGAGTTCGTGACCGCCGCC**
**GGGATCACTCTCGGCATGGACGAGCTGTACAAGTAA**GAATTCTCGCTAGATA
AGTAATGATCTTAATCAGCCATATCACATCTGTAGAGGTTTTACTTGCTTT*AAAAA*
*ACCTCCCACACCTCCCCCTGAACCTGAAACATAAAATGAATGCAATTGTTGTTGTTAACT*
*TGTTTATTGCAGCTTATAATGGTTACAAATAAAGCAATAGCATCACAAATTTCACAAATAA*
*AGCATTTTTTTCACTGCATTCTAGTTGTGGTTTGTCCAAACTCATCAATGTATCTTATCAT*
GTCTGGATCATCGATCGCGCGCAGATCTGTCATGATGATCATTGCAATTGGATCC
GGATAACTTCGTATAGCATACATCTATACGAAGTTATCCTAGGGGAATTCGACAT
CAAGCTTCTCAGG**ATGCCGGTGCGGAGGGGCCACG**
