# Supplementary figures and images for: Characterization of hERG1 channel role in mouse colorectal carcinogenesis
Source: Cancer Med. 2013 Jul 22;2(5):583–94. doi: 10.1002/cam4.72 (PMC3892791; doi:10.1002/cam4.72)

A

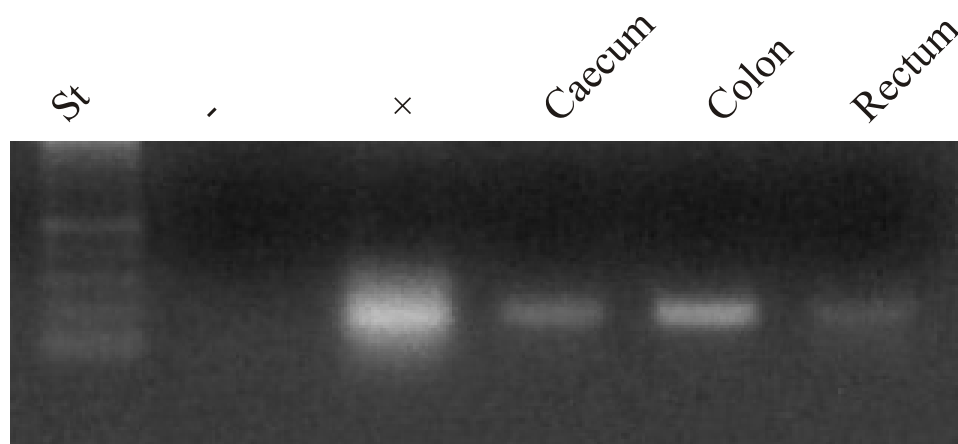

B

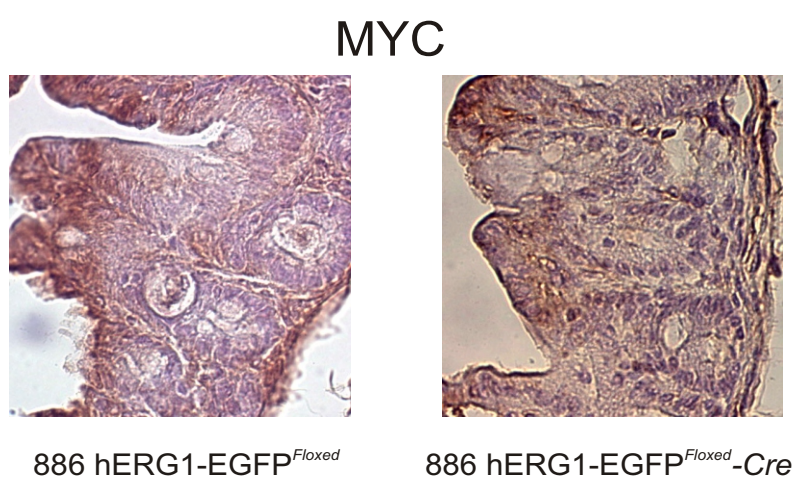

C

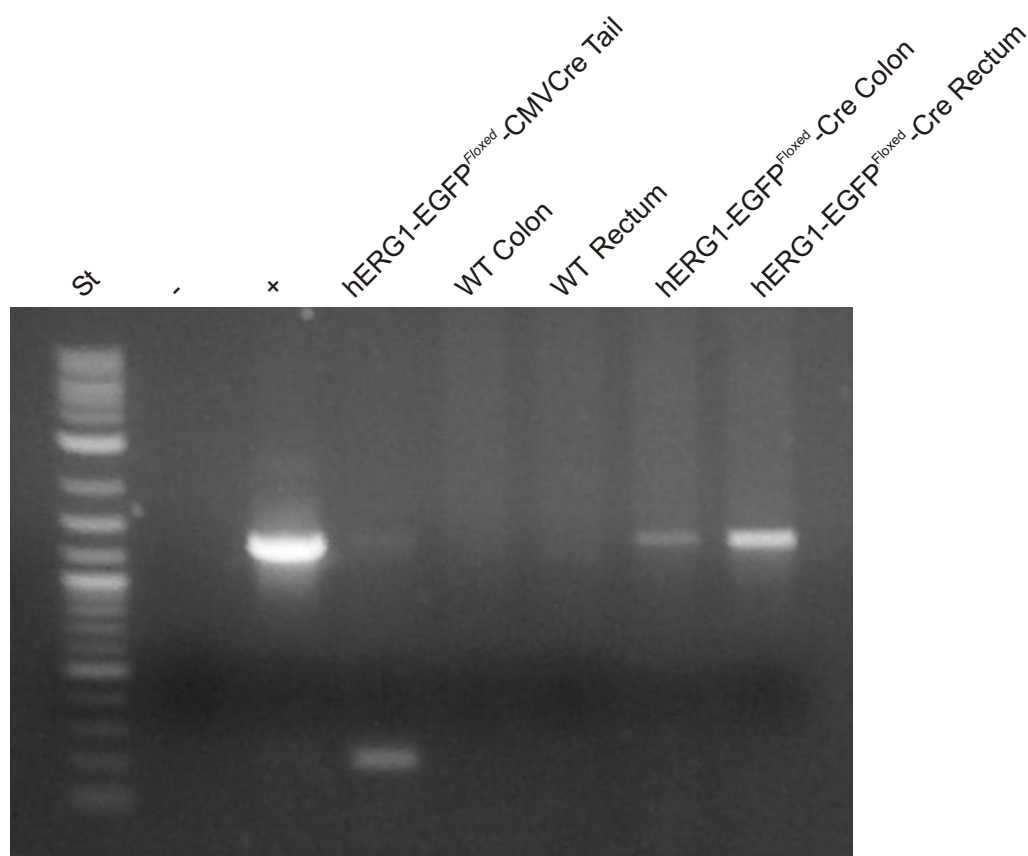

Supplement: Figure S2 — (A) hERG1-EGFPFloxed mice were mated with Fabp4xat-132Cre (Cre) mice. In the latter mice, the Cre recombinase should be expressed, although not exclusively, in the intestinal epithelium. Reverse transcription PCR showed mRNA expression of cre in different large intestine segment of hERG1-EGFPFloxed-Cre double transgenic (DT) mice. Amplification of cre was performed with Platinum _ PCR SuperMix on 2 lL of cDNA derived from cecum, colon, and rectum of DT mice and with a specific primer pair: 5′-ACCAGCCAGCTATCAACTCG-3′ and 5′-TTACATTGGTCCAGCCACC-3′ and applying the following PCR conditions: denaturation at 94°C for 2 min, 35 cycles at 94°C for 1 min, 60°C for 1 min, 72°C for 1 min, and a final extension cycle at 72°C for 3 min. (B) The lack of a significant difference in hERG1 expression in hERG1-EGFPFloxed-Cre compared to hERG1- EGFPFloxed mice was also confirmed at the protein level: an immunohistochemical analysis was carried out in colon-rectum of hERG1-EGFPFloxed and DT mice, employing anti-Myc antibody (anti-myc 9E10; Santa Cruz Biotechnology), dilution 1:100 in PBS-UltraVBlock). No gross difference in the amount of Myc staining could be detected in DT compared to hERG1-EGFPFloxed mice: hERG1-EGFPFloxed as well as DT mice showed myc expression in the stroma and in the epithelial lining. (C) hERG1-EGFPFloxed-Cre double transgenic genomic DNA was tested by end-point pcr to verify the presence of Cre mediated recombination in mice. DNA extracted from colon and rectum of WT and hERG1-EGFPFloxed- Cre double transgenic mice was amplified with the 5: 5′- AGGATCAGTCGAAATTCAAG-3′ and 4: 5′-ATGATGGTGTCCAGGAAG- 3′ primers and applying the following PCR conditions: denaturation at 94°C for 2 min, 35 cycles at 94°C for 40 sec, 52°C for 1 min, 72°C for 1 min, and a final extension cycle at 72°C for 5 min. Such end point PCR failed to detect recombination in hERG1-EGFPFloxed-Cre double transgenic mice (data not shown); on the other hand, PCR analysis performed on genomic DNA ex [file cam40002-0583-sd2.pdf]
